# COVID‐19 related anxiety in children and adolescents with severe obesity: A mixed‐methods study

**DOI:** 10.1111/cob.12412

**Published:** 2020-09-13

**Authors:** Ozair Abawi, Mila S. Welling, Emma van den Eynde, Elisabeth F. C. van Rossum, Jutka Halberstadt, Erica L. T. van den Akker, Bibian van der Voorn

**Affiliations:** ^1^ Obesity Center CGG, Erasmus MC, University Medical Center Rotterdam Rotterdam The Netherlands; ^2^ Department of Pediatrics, Division of Endocrinology Erasmus MC, University Medical Center Rotterdam Rotterdam The Netherlands; ^3^ Department of Internal Medicine, Division of Endocrinology Erasmus MC, University Medical Center Rotterdam Rotterdam The Netherlands; ^4^ Department of Health Sciences Faculty of Science, Vrije Universiteit Amsterdam Amsterdam The Netherlands

**Keywords:** COVID‐19, mental health, mixed‐methods, paediatric obesity, qualitative study

## Abstract

Recent studies report negative mental health effects of the COVID‐19 related lockdown measures in general paediatric cohorts. Since obesity is a risk factor for COVID‐19 in adults, children (including adolescents) with obesity might perceive themselves to be vulnerable. Using a combined quantitative and qualitative approach, we explored COVID‐19 related anxiety in paediatric patients with severe obesity in the Netherlands using semi‐structured telephone interviews and the Paediatric Quality of Life Inventory (PedsQL) questionnaire, which had also been completed by the study population at baseline in the year prior to the COVID‐19 outbreak. In total, 75 families participated in the semi‐structured telephone interviews during the lockdown, April 2020. Characteristics of included patients were: median age 10.5 years (interquartile range = 7.6‐15.2); 52% female; mean BMI standard deviation score 3.8 (SD = 1.0). COVID‐19 related anxiety was reported for 24/75 (32%) children. The mean decrease in PedsQL score between baseline visit and COVID‐19 outbreak did not differ between children for whom anxiety was reported vs those for whom it was not (mean change −10.3 ± 36.5 vs −3.3 ± 24.4, *P* = .54). Self‐imposed strict quarantine measures were taken by 19/75 (25%) families. During follow‐up, several families reported that the previous contact alleviated their anxiety. In conclusion, healthcare professionals should address possible COVID‐19 related anxiety in children with severe obesity. Addressing COVID‐19 related anxiety could mitigate its potential negative effects.

## 1 INTRODUCTION

During the current coronavirus disease 2019 (COVID‐19) pandemic, governments across the world have used differential lockdown and quarantine measures to mitigate the spread of the virus. Recent studies report how this situation affected the psychological wellbeing of children (including adolescents).^1^, ^2^, ^3^, ^4^, ^5^, ^6^, ^7^, ^8^, ^9^ These studies report several adverse effects on psychological wellbeing such as anxiety, worrying, irritability, depressive symptoms and even post‐traumatic stress disorder symptoms in 18.9% to 43.7% of children sampled from the general population in Asian, European or American countries. Moreover, a recent study in Italian children and adolescents with obesity showed unfavourable changes in eating, sleeping and activity behaviours during COVID‐19 quarantaine.^10^

Obesity is regarded as a risk factor for COVID‐19 in adults.^11^ Consequently, children with obesity might perceive themselves to be vulnerable. Moreover, we noticed COVID‐19 related concerns during our regular contacts with children and their parents at the outpatient clinic of our paediatric obesity centre when the governmental lockdown measures in the Netherlands were effectuated. On top of that, quality of life is already known to be diminished in children with severe obesity in comparison to the general population.^12^, ^13^ However, no studies have assessed such psychological aspects of the COVID‐19 outbreak in children and adolescents with obesity. Therefore, we designed a combined quantitative and qualitative study to explore the psychological impact of the COVID‐19 outbreak and related lockdown measures in children (including adolescents) with severe obesity and their potential effects on lifestyle behaviour. When conducting this study, COVID‐19 related anxiety appeared to be an important theme, similar to results from the previously mentioned literature from general populations. Accordingly, we want to present our in‐depth findings regarding COVID‐19 related anxiety in children with severe obesity and their parents.

## 2 METHODS

This study was approved by the ethics committee of the Erasmus MC. All data were collected for healthcare purposes and filed in the patient's medical records. Written informed consent was obtained from all patients and/or their caregivers to use their health data for research purposes after pseudonymization.

### 2.1 Study participants

In the Netherlands, selective lockdown measures including school closures were established from 16 March 2020 onwards. During the first month, between 2 April 2020 and 23 April 2020, when these measures were in full effect, we contacted all parents of children currently under treatment at Obesity Center CGG (Erasmus MC‐Sophia Children's Hospital), a national referral centre for obesity. Patients are referred to Obesity Center CGG for diagnostic evaluation and/or personalized therapeutic advice.^14^ We approached parents of all patients who had completed the diagnostic workup of our obesity centre and whose last visit to the outpatient clinic was in 2019 or 2020. We did not approach parents of children who have severe intellectual disability or severe behavioural problems, as we expected that their families' experiences during the lockdown period would not be representative. Because this study was conducted in the context of patient care, we included all eligible study participants even after data saturation for qualitative analyses had been achieved.

### 2.2 Telephone interviews

A semi‐structured telephone interview lasting 20 to 30 minutes, was conducted by a treating physician (OA, BvdV, MSW) to explore the impact of the COVID‐19 outbreak and related measures on the children's lifestyle behaviour and quality of life. In most cases, parents were interviewed as proxy for their children, and children were invited to actively participate in the interviews if verbal communication skills allowed it. All parents of eligible patients were contacted in a 3‐week time frame, during which the treating physicians had weekly meetings to discuss the previous' weeks findings and gain insights from each other's experiences. The physicians used a structured interview format with 37 predefined variables for categorical data and 20 predefined open‐ended questions to comprehensively document the telephone interviews in the patients' medical records. Additionally, field notes were collected during the interviews and qualitative analyses. The predefined interview question related to anxiety was: “Does your child experience stress or anxiety due to the Corona outbreak?” The predefined interview questions related to lockdown measures was: “What kind of lockdown measures did your family take, especially regarding: school? Day‐care attendance? Work? Social contacts? Hobbies?” Based on the answers on these questions, additional questions were asked in the context of patient care to further explore thoughts and reasons behind anxiety and imposed lockdown measures, and if present, whether our proactive support was necessary to minimize the impact on weight‐related health. After all interviews had been conducted, the comprehensive records were exported from the patient's medical records for analyses.

### 2.3 Quantitative assessments and analysis

Height and weight were measured during the previous hospital visit within the past year by trained outpatient clinic assistants and BMI was converted to age‐ and sex‐specific standard deviation scores (SDS) using Dutch reference charts.^15^ Both at the baseline visit prior to the COVID‐19 pandemic as well as during the lockdown measures, the 23‐item Paediatric Quality of Life inventory (PedsQL) 4.0 (parents proxy‐report version) was completed. We assessed the total score and the sub‐score for emotional functioning, ranging from 0 to 100 with higher scores indicating better quality of life.^16^ Quantitative data were analysed using SPSS version 25.0 (IBM). Differences in patient characteristics between patients for whom anxiety was reported compared to those for whom anxiety was not reported in the abovementioned question were analysed using (paired samples) *t*‐tests or Mann‐Whitney tests with an *α* of .05.

### 2.4 Qualitative analysis

Qualitative data were analysed using MAXQDA 2018 (VERBI Software) following best practice methods for qualitative studies and were reported following the Consolidated Criteria for Reporting Qualitative Research (COREQ) checklist.^17^, ^18^ Two physicians (OA, MSW) independently coded all interviews according to the Grounded Theory after all telephone interviews had been conducted.^19^ According to this theory, first a deductive, theory‐driven approach was used, followed by an inductive, data‐driven approach, by two of the three interviewing physicians. The two physicians started by open coding of interview data independently. The applied codes were then compared and differences were solved by consensus. Subsequently, a code tree was developed in a meeting with the study team using axial coding. To minimize the possibility of structural differences between the three physicians who conducted the interviews, the code tree was developed based on interviews from a subset of 24 patients, eight patients per interviewing physician. Finally, selective coding was used to identify the code categories that were most relevant to our research question. The axial and selective coding steps were also performed independently by both physicians and differences were solved by consensus. During the entire qualitative analysis process, a study log was kept by the two physicians and memos were used to carefully note emerging ideas about the data analysis which were discussed during weekly meetings with the study team, to further ensure rigour.

## 3 RESULTS

In total, 90 families were approached. Seventy‐five participated in the telephone interviews, of which 40 also completed the PedsQL questionnaire. Table 1 shows the baseline characteristics of the patients.

**TABLE 1:** Baseline characteristics of the study population

Characteristic	All patients (n = 75)	Children for whom anxiety was reported(n = 24)	Children for whom anxiety was not reported (n = 51)	*P* value
Age (y), median (IQR)	10.5 (7.6‐15.2)	11.0 (8.7‐15.9)	10.2 (6.8‐15.2)	.74
Sex, female (%)	39 (52%)	15 (63%)	24 (47%)	.21
Ethnicity, Dutch (%)	50 (67%)	17 (71%)	33 (65%)	.42
Socioeconomic status score, median (IQR)	0.0 (−0.7 ± 0.7)	0.0 (−0.6 ± 0.7)	0.0 (−1.2 ± 0.7)	.87
Body mass index SDS at last visit to hospital, mean (SD)	3.8 (1.0)	3.7 (0.9)	3.8 (1.0)	.87

Abbreviations: COVID‐19, coronavirus disease 2019; IQR, interquartile range; SDS, standard deviation score.

Anxiety related to the COVID‐19 outbreak and related measures was reported for 24/75 (32%) children. Baseline characteristics and quality of life did not differ significantly between patients for whom anxiety was reported vs not reported (Tables 1 and 2). The mean PedsQL total score between baseline visit and COVID‐19 outbreak slightly decreased in the study population, although not statistically significant (mean change −6.3 ± 29.9; *P* = .26). A bigger decrease was seen in the children for whom anxiety was reported vs those who did not (mean change −10.3 ± 36.5 vs −3.3 ± 24.4), but this was also not statistically significant (Table 2).

**TABLE 2:** Quality of life during COVID‐19 related lockdown measures

Characteristic		All patients (n = 40)	Children for whom anxiety was reported (n = 18)	Children for whom anxiety was not reported (n = 22)	*P* value^a^
PedsQL score on emotional functioning, mean (SD)	During COVID‐19	59.4 (21.8)	57.5 (24.0)	60.9 (20.3)	.63
	Delta baseline vs COVID‐19	−3.5 (35.2)	−5.0 (40.7)	−2.2 (30.7)	.82
PedsQL total score, mean (SD)	During COVID‐19	66.2 (17.7)	65.9 (20.0)	66.5 (16.2)	.93
	Delta baseline vs COVID‐19	−6.3 (29.9)	−10.3 (36.5)	−3.3 (24.4)	.54

*Note:* Baseline, measured at the outpatient visit in the year prior to the COVID‐19 outbreak.

Abbreviations: COVID‐19, coronavirus disease 2019; SD(S), standard deviation (score).

^a^ *P* value for the difference between children for whom anxiety was reported vs those who did not.

Table 3 reports the identified reasons behind this anxiety and the behavioural consequences. Most of the children with reported anxiety were afraid to be at increased risk for COVID‐19 infection. No children and only two parents specifically mentioned obesity as reason for their anxiety. In total, 19 families, either with children with reported anxiety (6/24; 25%) or without (13/51; 25%), took self‐imposed quarantine measures additional to governmental lockdown measures, such as total home confinement (Table 3). In five families with severe anxiety leading to negative lifestyle consequences, telephone follow‐up in the following weeks was deemed necessary in the context of patient care by the treating physician. During this follow‐up, 3/5 families reported that their concerns had been alleviated by information offered in the previous contact with the physician (Table 3).

**TABLE 3:** Identified themes regarding COVID‐19 related anxiety and lockdown measures and relevant passages from the documentation of the telephone interviews

Themes	Relevant passages
*Theme 1: Reasons for anxiety in children*	
Theme 1.1: anxious for being at risk for COVID‐19	Child (17 y, F) is afraid that she is more likely to get ill due to Corona because of her health problems. Child (10 y, M) is afraid he will get more ill than others from Corona.
Theme 1.2: anxious for health of family members at risk for COVID‐19 due to perceived vulnerability	Child (11 y, M) is concerned for his mother. He always wants to join her during her weekly visits to the supermarket. If it was up to him, she would stay home all the time. Child (9 y, M) is afraid his father might get ill, because his father has heart failure and COPD.
*Theme 2: Reasons for anxiety in parents*	
Theme 2.1: anxious for child being at risk for COVID‐19 due to perceived vulnerability	Mother is afraid that her child (5 y, F) is at increased risk because of her obesity. Therefore, they already confined themselves to home before governmental lockdown measures were taken. Father is not sure if he will let his son (11 y, M) go to school after school reopenings due to his asthma.
Theme 2.2: anxious for transmitting COVID‐19 to family members at risk	Child (15 y, F) is not allowed to have contact with friends, because parents fear she will transmit Corona to their 75‐year‐old grandfather who lives with them. Child (11 y, M) is not allowed to play with friends, because of his mother's asthma. He is also not allowed to visit his grandparents.
*Theme 3: Behavioural consequences of anxiety*	
Theme 3.1: additional restrictions imposed by parents regarding home confinement and social contacts	Parents cancelled all support and care from healthcare professionals on their own initiative because parents perceive their child (16 y, F) to be vulnerable. Initially, the family was anxious and stayed at home all the time. Yesterday mother and child (5 y, F) went outside for the first time since 3 wk. Child (11 y, F) is not allowed to play with friends anymore.
Theme 3.2: additional restrictions self‐imposed by child only	Child (11 y, M) is afraid to play outside. Even before the national lockdown measures were issued, he declined to go outside when his parents asked him to. In the past 1.5 mo., he only went outside three times. Child (9 y, M) does not want to meet with friends anymore, because he thinks his father is at increased risk for COVID‐19.
Theme 3.2: concerns alleviated by healthcare professional	In the beginning, the child (11 y, F) was afraid to be at risk because of her obesity. After the talk with healthcare professional X her concerns were relieved. Quote by mother of child (5 y, F): “For my own peace of mind, I will discuss my concerns with my general practitioner. I don't want to be afraid.”

Abbreviation: COPD, chronic obstructive pulmonary disease; COVID‐19, coronavirus disease 2019.

## 4 DISCUSSION

In this Dutch study, COVID‐19 related anxiety was reported for a considerable proportion (32%) of children with severe obesity under treatment at a tertiary centre. To our knowledge, this is the first study to investigate COVID‐19 related anxiety in children and adolescents with obesity, and only few studies explored similar psychological effects in children with other chronic diseases. A recent study in children with type 1 diabetes in India reported that moderate or severe stress was present in nearly 60% of their patients during the COVID‐19 pandemic, but this did not differ from age‐ and gender‐matched controls.^20^ Another study in children with cystic fibrosis in Turkey also did not find a difference in anxiety scores between their patients and age‐matched controls.^21^ In the general population, severe stress and traumatizing symptoms in children have been reported in a qualitative study from India and COVID‐19 related restrictions seemed to be the primary cause.^22^ This is in line with a previous qualitative report on the 2003 SARS and 2009 H1N1 pandemics, which showed that 30% of children who had been isolated or quarantined met the clinical cut‐off score for post‐traumatic stress disorder.^23^ These studies cannot be directly compared with ours due to differences in study population, design and sociocultural contexts. However, these studies together with ours imply that COVID‐19 related psychological distress such as stress and anxiety might be experienced by a significant minority of children and adolescents, both with and without obesity.

Recent reports show that lifestyle behaviours including physical activity and screen time are negatively impacted by the COVID‐19 outbreak and related lockdown measures in Chinese school children and Italian children with obesity.^10^, ^24^ In a significant proportion of the families (25%) in our study, self‐imposed quarantine measures were taken, even though measures advised by our national authorities did not differentiate between children with obesity or other chronic diseases and healthy children. These strict self‐imposed measures are a concern because they can add to the known negative effects of the COVID‐19 pandemic on lifestyle behaviour. The anxiety that potentially underlies these self‐imposed measures seems to be modifiable. In the families for whom short‐term follow‐up was necessary, we experienced that discussing this emotion with patients and parents and educating them can relieve concerns and make them lift their strict self‐imposed measures. Topics that can be discussed with parents and children, using age‐appropriate language, are: reassurance that children with obesity are currently perceived to be at low risk; reduction of exposure to COVID‐19 related (social) media outlets; maintaining daily life routines as much as possible given governmental measures; encourage children to maintain social contacts, for example, via the internet and stimulating parents to promote positive mental and social wellbeing in their families and involving their children in the process.^25^ Our qualitative analysis indicated that two important reasons behind the anxiety were the child's fear of being at risk for COVID‐19 and the fear of infecting family members who are perceived to be vulnerable for COVID‐19. In addition, the recent report on patients with cystic fibrosis found, similar to us, that anxiety could be alleviated in 84% of mothers by the healthcare professional during a telephone interview.^21^ It is known that worrying of children for their parents can put a heavy burden on them, and effective communication with children can protect their psychological health.^26^, ^27^ We did not find differences in baseline characteristics nor in quality of life assessed by the PedsQL questionnaire or obesity severity between patients with and without COVID‐19 related anxiety. This underscores that healthcare professionals should be aware of the possible presence of COVID‐19 related anxiety during all contacts with children and adolescents with severe obesity, not only in specific subgroups.

### 4.1 Strengths and limitations

A strength of our study is our qualitative approach which enabled us to explore possible arguments behind COVID‐19 related anxiety and its potential modifiability. Moreover, our relatively large sample size allowed us to reach data saturation. A strength of our quantitative analyses is the comparison of PedsQL scores before and during the COVID‐19 outbreak, as it is known that quality of life is already compromised in children with severe obesity.^12^, ^13^ A limitation of this study is its cross‐sectional analysis; follow‐up studies are needed to evaluate the course and effect of COVID‐19 related anxiety on weight‐related health and will be performed for our patient group. We did not consider including a control group without obesity because our study was designed to explore the impact of the COVID‐19 outbreak and its consequences on lifestyle behaviours specifically in children with severe obesity. Accordingly, our patients served as their own control for the quantitative analyses. This should be kept in mind when attempting to extrapolate our findings.

In conclusion, healthcare professionals should be aware of the possible presence of COVID‐19 related anxiety and its behavioural consequences, especially in children with severe obesity. Addressing this anxiety could mitigate its potential negative effects on the psychological wellbeing and lifestyle behaviours of these children.

## CONFLICT OF INTEREST

No conflict of interest was declared.
